# Molecular Aspects of MicroRNAs and Phytohormonal Signaling in Response to Drought Stress: A Review

**DOI:** 10.3390/cimb44080253

**Published:** 2022-08-16

**Authors:** Hafiz Muhammad Ahmad, Xiukang Wang, Munazza Ijaz, Mahmood-Ur-Rahman, Sadaf Oranab, Muhammad Amjad Ali, Sajid Fiaz

**Affiliations:** 1 Department of Bioinformatics and Biotechnology, Government College University, Faisalabad 38000, Pakistan; 2 College of Life Sciences, Yan’an University, Yan’an 716000, China; 3 Department of Biochemistry, Government College University, Faisalabad 38000, Pakistan; 4 Department of Plant Pathology, University of Agriculture, Faisalabad 38000, Pakistan; 5 Department of Plant Breeding and Genetics, The University of Haripur, Haripur 22620, Pakistan

**Keywords:** drought stress, microRNA, phytohormones, gene expression, stress regulation

## Abstract

Phytohormones play an essential role in plant growth and development in response to environmental stresses. However, plant hormones require a complex signaling network combined with other signaling pathways to perform their proper functions. Thus, multiple phytohormonal signaling pathways are a prerequisite for understanding plant defense mechanism against stressful conditions. MicroRNAs (miRNAs) are master regulators of eukaryotic gene expression and are also influenced by a wide range of plant development events by suppressing their target genes. In recent decades, the mechanisms of phytohormone biosynthesis, signaling, pathways of miRNA biosynthesis and regulation were profoundly characterized. Recent findings have shown that miRNAs and plant hormones are integrated with the regulation of environmental stress. miRNAs target several components of phytohormone pathways, and plant hormones also regulate the expression of miRNAs or their target genes inversely. In this article, recent developments related to molecular linkages between miRNAs and phytohormones were reviewed, focusing on drought stress.

## 1. Introduction

Abiotic stresses are common problems of every ecosystem and rely on various environmental factors [1,2]. Drought is among the most hazardous stresses among all other abiotic stresses that affect crop plants throughout the world [3,4]. A recent study has revealed that the total yield loss triggered by drought is approximately 7% worldwide [5,6]. It was estimated that by 2050, water stress will severely affect cultivated land and ultimately affect the two-thirds of the global population [7,8]. Drought affects crops in different ways, and even crops with the same drought tolerance might have different gene expression and metabolism. As a result, identification of drought-tolerant genetic resources and determining the best technique to avoid crop loss are critical [9]. Adverse effects of drought can be eliminated by post-transcriptional regulation of genes associated with signal transduction, protein biosynthesis, energy metabolism, photosynthetic activity, and membrane trafficking [8,10]

Phytohormones are key signaling molecules responsible for all biological and metabolic regulators in the plant’s life cycle [11]. Critical phytohormones include ethylene (Et), abscisic acid (ABA), salicylic acid (SA), cytokinin (CK), gibberellic acid (GA), auxin (Aux), indole acetic acid (IAA), brassinosteroids (BR), jasmonic acid (JA), and strigolactones (SL) [11,12]. Among these plant hormones, five hormones (ABA, IAA, CK, GA and ET) are classical hormones, whereas the rest (JA, SA, BR and SL) are recognized as putative or growing phytohormones. In plants, phytohormones are synthesized via various pathways and are perceived by specific receptors, triggering intracellular signal transduction [12]. Phytohormones work together to alter multiple cellular processes, such as elongation of cells, vascular root patterning, and management of abiotic and biotic stress response.

Intracellular phytohormones are important growth regulators, but they have significant functions in plant drought stress tolerance by regulating physiological processes and molecular interactions [13]. Drought stress has an impact on phytohormone generation, accumulation, and distribution throughout the plant body [13]. After signal perception, ABA is the most critical phytohormone and the most important hormone produced in response to water scarcity [14,15]. Jasmonates have a key function in fruit ripening, root growth, and reproduction. This hormone also plays an important function in regulating plant water stress reactions [13]. Salicylic acid (SA) was established by several researchers to play a significant function in plant under drought stress [13]. Low amounts of SA boost plant antioxidant capability, but greater levels can kill cells or make plants more vulnerable to abiotic stress [11]. SA stimulates genes with roles in the production of secondary metabolites, chaperones, organic acids, and heat shock proteins [16,17]. Cell elongation, vascular tissue improvement, and apical dominance are all known impacts of indole-3-acetic acid on plant elongation and development [18]. During drought conditions, IAA appears to assimilate plant development and upregulate gene expression linked with root meristem initiation, enhancing root branching, and increasing plant stress tolerance [13,18].

Plant microRNAs (miRNAs) are 20–24 nucleotide-long post-transcriptional regulatory molecules [19,20]. These highly conserved molecules play a function in various systems of mature plants, including plant growth, development, and stress tolerance. miRNA is a small RNA that control the expression of different genes involved in biological and metabolic processes. Because it induces divergence in the expressed gene, the interaction between miRNA and its mRNA target is particularly significant [21]. A single miRNA can target various additional genes in the same cell signaling pathway. Through endonucleolytic cleavage or translational inhibition of mRNA cognate targets, miRNAs operate as a negative gene expression regulator [19,22].

Stress response modulation via the miRNA pathway was found in several plant species [23,24]. To combat with severe environmental conditions, plants have evolved many strategies for modifying the expression of genes that regulate physiological processes. Drought stress was demonstrated to boost plant miRNA expression [25]. It has an impact on several biological processes, including stem, root, leaf, and flower production. In response to miRNA regulation, plants are affected by biotic and abiotic stress, hormone signaling, and nutritional balance [26].

Plants naturally have different processes to acclimatize to changes in harsh conditions, including pathways in which miRNAs play a vital role in biotic and abiotic stress conditions. miRNA genes are up- or downregulated in a variety of species, including soyabean (*Glycine max*), sugarcane (*Saccharum officinarum*), rice (*Oryza sativa*), and maize (*Zea mays*), under different stresses [24,27,28]. Multiple evidences are available in literature that indicate the expression or accumulation of these miRNAs to further explain the regulatory networks connected with stress defense mechanisms [23]. Several miRNAs have critical role in the morphological development of the fruit. miR164, miR156/miR157, miR396, and miR160 expression, for example, can cause abnormal fruit, such as fused carp and reduced fruit size and morphology [29,30].

This review focused on the recent knowledge about phytohormones, miRNAs, and their involvement in responses to drought stress in crop plants. We have tried to outline the impacts of miRNAs and phytohormones on the expression of drought-related genes. Another section addresses the crosstalk among miRNAs and plant hormones during drought conditions. At the end, we discuss about the potential of miRNAs to improve abiotic stress, such as drought, tolerance in crop plants.

## 2. Drought-Responsive Genes Are Regulated by Plant Hormones

Plant hormones are chemical messengers that control growth, development, and metabolic activities in plants under different biotic and abiotic stimuli [31]. Plant survival is strongly linked to hormone-mediated main regulatory mechanisms, which is a complicated process involving multiple interactions at the transcriptional, translational, and cellular levels [10,16]. Plant hormones and miRNA-mediated gene regulation are critical regulators of gene expression in both normal and stressful situations [32]. Plants response to environmental stress differs due to variations in phytohormone levels.

Plant hormones, such as abscisic acid (ABA), auxins (IAA), gibberellin (GA), cytokinin (CTK), salicylic acid (SA), and jasmonic acid (JA), regulate the drought stress in plants [10,33,34]. However, the mode of action of various phytohormones to eliminate/escape the drought stress is diverse, depending upon the developmental stage, plant tissues and drought prevailing conditions. During drought stress, some plant hormones help modify the root architecture of plants, influence the photosynthetic machinery, modulate the water balance, enhance the antioxidant defense system and control the drought-related gene expression in plants [10].

Abscisic acid (ABA) is a crucial phytohormone that have a significant role in regulating different signaling pathways under environmental stresses [35,36]. During drought stress, ABA accumulates in guard cells via ABA biosynthesis pathway. ABA synthesis reduces turgor pressure and causes the closure of stomata, decreasing the transpirational water loss [37]. ABA is absorbed in plant leaves at the morphological level, improving cell wall extensibility, tissue turgidity, and root hydraulic transmission [36]. ABA content enhances drought resistance by improving total chlorophyll contents, more stem dry weight and high regulation of drought-tolerant genes such as *RD22*, *RD29B* and *bZIP* [33]. ABA regulates root growth to reach the deep water in the soil during osmotic stress. Interaction of ABA with other plants hormones leads to developing the lateral roots in plants necessary to tolerate dehydration stress [33]. Accumulation of ABA during drought conditions was observed in wheat, rice, sorghum, barley, and soybean [35,38].

ABA influences and controls the regulation of several genes by forming osmoprotectants and defensive proteins [35]. Upregulation of rice guard cell genes (SNAC1) enhances ABA sensitivity, drought stress tolerance, and closure of stomata [39]. Overexpression of *OsbZIP72* and *OsbZIP46*, which boosted the expression of ABA sensitive genes, improved rice drought tolerance considerably [40]. In rice plants, upregulation of *OsMYB48–1* boosted the expression of genes produce ABA such as *OsNCED4* and *OsNCED5* [41]. *GmHP08* improves drought tolerance in soybeans via ABA-dependent pathways [42]. Drought resistance in *Arabidopsis thaliana* is improved by overexpression of the *AtSAUR32* gene, which accumulates ABA and IAA hormones [43]. By inducing ABA and ROS scavenging, *Arabidopsis* ascorbic acid peroxide genes such *AtAPX2* increased water usage efficiency and drought resistance [44,45].

Auxin/Indole 3-acitic acid (IAA) was the first phytohormone discovered to have important functions for plant growth and development through cell elongation, tissue differentiation, axial elongation, and apical dominance [36]. Auxin regulates all aspects of plant life, from embryogenesis through senescence [32] although an increase in auxin levels was linked to a decrease in growth, indicating that a change in hormonal balance is to blame for a decrease in growth under stress [32,37]. Auxin also promote roots branching, potentially significant for increasing drought tolerance [46]. Studies proved that miRNAs could control the auxin signal transduction, and on the other hand, various auxin signaling genes were observed to target the miRNAs. In addition to genes, some auxin-responsive factors (ARFs) were reported as miRNA targets, i.e., ARF10, ARF16 and ARF 17 were upregulated by miRNA160 and miRNA167 where they were downregulated by ARF2, ARF3, ARF6 and ARF8 [47]. Activation of the *OsGH3-2* gene encoding the IAA inactivation enzyme decreases free IAA content and various changes in the pattern of drought resistance in transgenic white clover (*Trifolium repens*) [33]. Drought stress was observed to upregulate two *OsPIN* rice genes, *OsPIN2* and *OsPIN5b* [48]. *TLD1/OsGH3* overexpression enhances the expression of LEA genes, resulting in greater drought tolerance in *Oryza sativa* plants, suggesting that auxins activate a number of interrelated metabolic pathways [49].

According to previous study, miRNAs could be a suitable pathway for incorporating auxin. Auxin genes such as *OsIAA6* were found to be induced by drought stress and are involved in dehydration stress resistance [50]. In white clover, upregulation of auxin and drought-responsive genes such as *bZIP11, MYB14, MYB48, DREB2, GH3.1, GH3.9, IAA8, WRKY2, WRKY56, WRKY108715*, and *RD22* was found [33]. *OsGH3-2,* an auxin and ABA regulating gene, was implicated in rice drought stress regulation [51].

Ethylene is a methionine-derived metabolite that is sensitive to biotic and abiotic stressors. Stage ethylene controls shoot growth, stem thickness, root elongation, stomatal density, and leaf abscission at the seedling level [52]. It has, however, been extensively researched in the process of plant senescence, although it is less well understood in drought-induced senescence [53]. Under drought stress, ethylene was proven to cause leaf abscission, which reduces water loss [54]. Water stress stimulates the de novo synthesis of 1-aminocyclopropane-1-carboxylate (ACC) synthase, an ethylene biosynthesis rate-controlling enzyme [53]. Furthermore, ethylene and its metabolic process induce plant responses to flooding and water scarcity, and it is involved in a variety of abiotic stress-related plant metabolic activities [55]. Drought stress tolerance/resistance mediated by ethylene was seen in (*Glycine max*), rice (*Oryza sativa*), and maize (*Zea mays*), potato (*Solanum tuberosum*), and *Arabidopsis* plants [56]. Rice proline synthesis and drought tolerance have both been linked to *OsEBP89*, a member of the AP2/ERF family [55]. The gene *SlERF36* was found to play a role in stomal density, plant growth, and photosynthetic activities in potato (*Solanum tuberosum*) plants [57].

The phytohormone ethylene has a regulatory role in root elongation under drought conditions by interacting with auxin. During the seedling stage in rice plants, ethylene interacts with the auxin biosynthesis gene *OsELI1* to inhibit the enlargement of roots [58]. A mutant *etol1* was identified in *Arabidopsis* and rice, which accumulates more ethylene ad affect the stomatal closure and ROS production in guard cells [59].

The plant hormone cytokinin (CK) was first found in maize crop. Plants produce CKs in their root tips, which are then transferred to the xylem. Cell division, vascular and shoot differentiation, nutrient mobilization, anthocynin production, photomorphogenic development, and leaf senescence are all pathways in which CK is required [60,61]. CK was reported to trigger in response to drought stress and exert positively or negatively on drought regulation, depending on stress interval and frequency in plants [10].

CKs are considered a crucial regulator of root length, root branching; hence it plays a significant role for elongation of primary roots and branching in response to drought stress [62]. Transgenic cotton (*Gossypium hirsutum*) enriched isopentenyl transferase (IPT) expression has resulted in higher endogenous CK levels, deferred plant senescence and enhanced drought tolerance [63]. CK improved drought tolerance in the transgenic barley lines through overexpressing the CK dehydrogenase gene [64].

The transgenic roots of barley also demonstrated significantly greater auxin productivity. Functional analyzes of CK receptor mutants revealed that certain *Arabidopsis* and *Brassica napus* CK receptors, namely *AHK2, AHK3, CRE1/AHK4, BnCHK1 and BnCHK3, and BnCHK5* perform as negative osmotic stress regulators [65]. *ARR1*, *ARR10*, and *ARR12*, *Arabidopsis* type B CK response regulators, were shown to promote drought stress tolerance by improving cell membrane integrity, ABA hypersensitivity, anthocyanin production, and stomatal aperture reduction [66]. Similarly, type A CK response regulators (*AHK2*, *AHK3* and *AHK4*) expressed higher ABA sensitivity and drought stress tolerance [67,68]. The higher expression of CKs from type B triple mutants arr1, arr10, and arr12 suggested that they play a role in ABA–CK crosstalk and were triggered by water stress [44,66].

Plants produce salicylic acid (SA), a phenolic molecule that works as a growth regulator and controls plant maturity, and abiotic interactions [69,70]. According to current research, SA plays a critical role in plants under different abiotic stresses such as drought stress [34,71]. SA improves drought resistance through influencing plant physiological systems such as photosynthetic activity, the production of antioxidants, and stress tolerance genes [70].

SA application in *Arabidopsis* regulates *the ICS1* gene and confirms the drought tolerance. Many WRKY TFs such as *WRKY70* and *WRKY54* are widely distributed among plant species are governed by SA application [72,73]. Overexpression of *WRKY70* TFs regulates *HD-zip-I* genes under limited water conditions [72]. *WRKY70* and *WRKY54* cooperate as a negative regulator of osmotic stress tolerance and stomatal closure in *Arabidopsis,* showing their importance in abiotic stress signaling [73,74]. Drought resistance in rice, tomato, wheat, and bean (Phaseolus sp.) plants was confirmed by exogenous SA treatment [75,76]. A putative method for maintaining the water content in plant leaves was suggested: SA-induced stomatal closure [75]. AP2/ERFs, leucine zipper, Zn fingers, and other transcription factors, according to gene expression profiling studies, have responded to SA and drought stress, showing that this phytohormone plays a substantial role in drought stress response. In *Arabidopsis,* SA-dependent miRNA167 influenced flower and root growth as well as osmotic stress resistance [77].

Jasmonic acid (JA) is an important phytohormone produced in the chloroplast, cytoplasm, and peroxisomes. JA and its derivatives are known as jasmonates with significant roles in biotic and abiotic stresses in plants [2]. JAs are widely found in plant blooms [34,69]. This plant hormone regulates development and is involved in flowering, fertilization, and main root growth [34]. During drought stress JA enhances water uptaking capacity of pants by altering root hydraulic conductivity [78]. JA moderate the effect of low moisture conditions by regulating the signaling of secondary metabolites. Moreover, JA improves antioxidant activities by enhancing the production of osmoprotectants and compatible solutes [79]. The first ever response of JA against dehydration stress was reported in barley, where it was noted that an increased level of JA enhanced the transaction induction of relevant downstream genes [80].

Drought tolerance mediated by JA has since been found in wheat [79], rice [81], maize [82], and chickpea (*Cicer arietinum*) [83]. The major precursor of JA, 12-OPDA, is involved in stomatal closure regulation in a model plant [84]. The gene *OsbHLH148* interacts with *OsJAZ* in rice plants under drought stress, and high levels of *OsDREB1* expression promote drought tolerance [37]. Another study found that upregulation of the OsbHLH148 gene makes rice plants more resistant to desiccation stress [85]. According to the findings, JA is involved in plant defense not only during injury and pathogen attack, but also during drought. Despite a lot of research on the role of JA in drought tolerance, more research is needed to fully understand drought tolerance in plants [37].

Gibberellic acids (GA) are important plant hormones which are involved to control the growth-related traits, i.e., cell expansion, cell elongation, leaf, stem, root and fruit growth [71]. Plants synthesize various GAs but GA1 and GA4 are the most active types of these hormones [37]. These phytohormones respond to drought stress by affecting photosynthetic enzymes, nutrient use efficiency, leaf area index and stomatal conductance [71]. GAs are synthesized in cytoplasm, plastids and endoplasmic reticulum [53]. Application of GAs to improve low moisture stress tolerance was observed in maize [71,86] wheat [87] and sunflower [88]. The *Arabidopsis* methyl transferase gene 1 *AtGAMT1* was overexpressed in tomato plants to create drought-resistant transgenic tomatoes [37]. It was also found in another study that the GA 2-Oxidase gene (*OsGA2ox*) was found to improve rice drought resistance [89]. In a recent study, soybean TF *GmTGA15* was shown to be overexpressed in plants with low moisture content [17].

Brassinosteroids (BRs) belong to a novel group of steroidal plant hormones that play role in morphological and physiological changes of plant by altering their growth and development [90,91]. Plant BRs are synthesized in immature seeds, pollens, flowers and roots and play indirect role to modulate the drought by stimulating the H_2_O_2_ level of plants [92,93]. In *Arabidopsis*, wheat, clover, tomato, and brassica, BRs have depicted positive functions under drought stress [94,95]. BRs interact with other plant hormones, such as ABA, to reduce the severity of drought stress in tomato plants [91]. BR precursor’s 24-Epibrassinosteroid and 28-homobrassinolide include favorable modifications in *Arabidopsis*, brassica, purple mustard, and pepper (*Capsicum annuum*) plants’ photosynthetic antioxidant system during restricted water stress [90,96]. Overexpression of the *Arabidopsis* BR biosynthesis gene *AtDWF4* in canola boosted growth, yield, and water stress tolerance, according to research [97]. Furthermore, whereas RNA interference improved drought stress tolerance in Brachypodium [98], overexpression of the *SlBRI1* gene reduces drought tolerance in tomato plants, suggesting that drought tolerance is reliant on defective BRs production pathways. [91,95]. The genes *AtDREBD2A* and *AtNCED3* were elevated in *Arabidopsis* plants after exogenous BR treatment [90]. Various phytohormones that regulate drought responsive genes are listed in Table 1.

The BR-related gene *OsLAC*, which is connected to grain yield during osmotic stress, is inhibited by upregulation of miR397 in *O. sativa* and *Arabidopsis.* [99,100]. Strigolactones (SLs), which are generated from carotenoids, are a recent addition to the phytohormones family [101]. SL, a carotenoid-derived terpene lactone, was isolated from a *G. hirsutum* root culture solution in the 1960s [102]. Under environmental limits, SLs are biosynthesized at the plant root and induce the production of lateral roots and root hairs to increase the absorption of restricted inorganic nutrients by the roots [101]. Simultaneously, these SLs are transported to above-ground plant sections, limiting the growth of lateral buds or branches and lowering the branches’ inorganic nutritional requirements. Because they are generated in plant roots and transported to the rhizospheric zone, these plant hormones are best known for their role in the rhizospheric zone. The primary types of SLs analogues that are chemically produced are GR5, GR7, and GR24 [93,101]. Recently, the functions of SLs in reducing the negative effects of abiotic stressors were documented [93]. In *Arabidopsis* exogenous GR24 has several regulatory functions for drought tolerance. When drought stress was applied to SL depleted *Arabidopsis* and tomato mutants, plants revealed alterations in stomata and ABA levels. When lettuce plants were drought stressed, the *Arbuscular mycorrhiza* symbiosis changed the level of SLs in the root systems, according to other studies [103]. SLs were found to initiate and control stomatal closure in response to stressors, and the corresponding molecular mechanism for controlling stomatal closure was also elucidated [104]. Drought tolerance in *Arabidopsis* was proven to be positively regulated by strigolactone *DWARF14*, which modulates abscisic acid response, cell membrane integrity, accumulation of epicuticular waxes, stomatal closure and biosynthesis of anthocyanin [105].

## 3. Drought Stress Regulation by miRNAs in Diverse Crop Species

**Figure 1:**
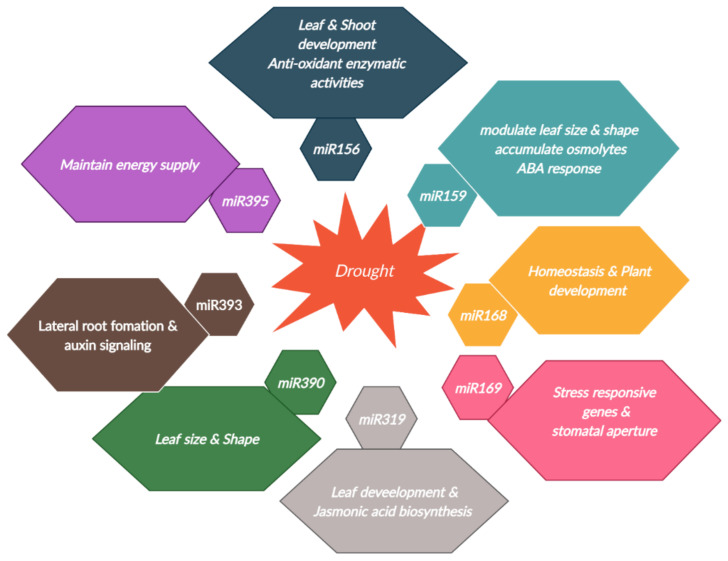
Functions of miRNAs in response to drought stress in crop plants.

Physiological, molecular and transcription levels of drought tolerance were well characterized in crop plants [3,122]; however, miRNA mediation has not yet been properly explained [11]. In addition to plant development, miRNAs also regulate abiotic stress-responsive genes in plant species [123]. So, by understanding the mechanism through which miRNAs respond to stress-responsive genes and which genes are the targets of miRNAs will help to develop more resistant plants [124,125]. As an important regulator of the plant regulatory network, prior importance was given to miRNAs for post-transcriptional regulation of drought tolerance [125]. Due to their salient features, drought-responsive miRNAs were characterized in *Arabidopsis,* cereals and oil seed crops [126,127,128,129,130]. Role of various miRNAs to overcome the drought tolerance traits has been shown in Figure 1. A study about the miRNA transcriptome using high-throughput sequencing technologies in wild barley may be an effective way to determine the drought resistance attributes of miRNAs and their target genes [9]. When maize plants were subjected to drought stress, various miRNAs were upregulated, and some miRNAs were downregulated [131]. Drought stress also upregulated the expression of miRNAs in wheat [132] and rice [133,134]. In *Arabidopsis,* the gene expression of miR393, miR397, and miR402 increased, while the expression of miR319c and miR389a decreased under dehydration stress [99]. miR398 and miR408 are thought to induce drought tolerance in pea (*Pisum sativum*) [135] and clover [136]. In tomato plants, miR159, miR169, miR160, miR167, miR393 are associated with dehydration stress tolerance, by controlling hormonal signal transduction, stomatal closure and auxin-responsive genes [137,138]. miR164 was expressed in the leaf and roots of orchard grass when these plants were exposed to dehydration treatments [139]. A significant decrease in the expressions of miR530a, miR1445, and miR1447 in *Populus trichocarpa* was observed in plants under limited water stress, which varies from the expression pattern of miR1450 under drought conditions [140]. Similarly, when sugarcane plants were subjected to drought, several miRNAs showed higher expression and some were downregulated [141]. Further, it was observed that the expression pattern of miRNAs is also plant tissue growth stage and cultivars dependent. ABA treatment in rice downregulates the expression of miR167 [142]; however, drought stress upregulates it in *Arabidopsis* [143]. miR169 was downregulated in *Arabidopsis* and clover during drought, while it was upregulated in rice [144]. Drought stress reduced the expression of miR398 in maize [130], whereas its expression increased in clover [144].

## 4. Crosstalk between Plant Hormones and miRNAs during Drought Stress

Hormone signaling and gene expression possess probabilistic roles in plant growth under miRNA control [145]. The first link between miRNAs and phytohormones (ABA, IAA and CK) was observed in the *Arabidopsis* plant [146]. After that, it was also confirmed that GA controls miR159 during anther development. [147]. In addition, during *Arabidopsis* seed germination, it was revealed that miR160 promotes the production of the auxin response transcription factor *ARF17* [148] and that miR159 induces ABA to bind to MYB (*MYB33* and *MYB101*) mRNAs [149]. Many mRNAs involved in hormonal reactions, such as *TIR1* and negative auxin signaling, are likely targets for miRNAs, and recent research has shown that *TCP* (TEOSINTE BRANCHED/CYCLOIDEA/PCF), the miR319 target, regulates the biosynthesis of the hormone jasmonic acid [150]. Despite the fact that the expression of miRNAs in plant hormone signaling is still poorly understood, no miRNAs were linked to cytokinin or ethylene signaling [142]. Three miRNAs, miR162, miR167, and miR413, are controlled by ABA during environmental stress and are responsible for stress tolerance and stress-related gene expression [142]. Furthermore, GA signaling downregulates two miRNAs, miR166 and miR319, which confer drought stress tolerance in plants [142]. According to available research, ABA regulates miRNA expression and influences the expression of some miRNA. For example, ABA regulates the expression of miR159, miR169, and miR172 in the embryonic callus of the Japanese larch (*Larix kaempferi*) [125].

In *Arabidopsis*, the higher gene expression of miR160 reduces ABA sensitivity during germination and tends to cause unusual root morphology, leading to the promotion of adventitious roots and lack of gravitropic responses [151]. During osmotic stress overexpression of miR172b reduces leaf water loss, increased ABA sensitivity and increase survival rate in soybean and *Arabidopsis* [152]. Researchers suggested that miR394 is implicated in ABA or ABA-dependent drought reactions in *Arabidopsis* [153]. Drought resulted in the miR393-dependent regulation of AUX signaling by the downregulation of *AtTIR1* and *AtAFB2* genes, which are growth inhibitors and associated with increasing ABA levels [154]. The overexpression of miR393, which inhibits the expression of *OsTIR1* and *OsAFB2*, causes alterations in rice root development and drought tolerance [155].

Some miRNA expression is also affected by exogenous ABA, with miRNA controlling the downstream genes of ABA. ABA hypersensitivity and drought tolerance increase on the overexpression of miR168a, while hyposensitivity and dry hypersensitivity are observed in knockout miR168a-2 [156]. In *Arabidopsis*, ABA promotes the expression of mature miR394 and precursor miR394a/b [156]. This is also linked to the overexpression of miR396 in plants, which is responsible for reduced root length [157]. It has also been suggested that miR396 expression influences root expansion via the ABA/ET pathway. Because it was demonstrated to influence the expression of numerous ethylene response factor (ERF)- and ABA-related genes, miR396-GRF regulation of the ET and ABA pathways may have persisted [156]. Drought tolerance has also been enhanced by AtmiR396a and AtmiR396b by influencing morphology of leaf [142]. Draught conditions in plants are thought to be influenced by miR396 in ABA-mediated plant adaptation. Overall, the findings revealed that miR396 has a key role for control of cell propagation by ABA and ET in response to abiotic and biotic stresses [157].

Transgenic plants expressing the miR160-resistant *AtARF10* form demonstrate the higher expression of several ABA-regulated genes as well as dose-dependent hypersensitivity to ABA, pointing to AUX as a possible ABA response modulator. ABA reduces the expression of miR167 in rice seedlings [142]. When this regulation occurs during germination, the production of miR167 is increased due to miR160′s suppression of the ABA pathway, which promotes lateral root formation by *IAR3* depression caused by AUX [53]. When rice seedlings were exposed to ABA treatment, the expression of miR167 was significantly reduced [142]. Similar regulation during the germination stage may restrict the ABA pathway, causing miRNA160 and miR167 production to increase. Despite the fact that ABA inhibits *LCR* (*LEAF CURLING RESPONSIVENESS),* which is a target of miR394a/b, and overexpression of miR394a/b results in ABA hypersensitivity and ABA-related phenotypes, overexpression of *LCR* results in ABA-resistant phenotypes [153]. Role of miRNAs via plant hormones to control the drought related genes/traits in various plant species is listed in Table 2.

Furthermore, wild-type and *LCR* overexpressing plants collect more ABA-induced hydrogen peroxide and superoxide anion radicals than miR394a/b-expressing plants [153]. Drought-responsive miRNAs were found in barley [142]. Under dehydration stress, *Arabidopsis* showed upregulation of miR393, miR397b, and miR402, but downregulation of miR319c and miR389a [99]. It was discovered in another study that during dehydration stress in rice seedlings, 17 miRNAs were downregulated, including miR164c, miR319b, and miR1861d, while 16 miRNAs (miR166h, miR172d, miR408, and others) were upregulated [128]. MiR398 and miR408 are downregulated in pea under restricted water stress [135]. The crosstalk and co-expression network of miR396 and miR397 reveal a link between BR sand auxin in rice growth and yield control [158]. Gibberellin (GA), which accumulates with BR, can trigger elongation by interacting with miRNAs and target genes such SCR (Scarecrow), DELLA, and GRF. BR affects rice yield via interacting with auxin and/or GA [159]. Depending on the abiotic stress, different miRNAs influence the expression of phytohormone-related genes. In response to drought stress, slymiR160, slymiR2199, and slymiR6426 targeted the stress response gene ARF, which is associated with auxin signaling. After selenium treatment in Astragalus, however, miR167 targeted auxin-responsive factor (ARF) [134]. Drought resistance is aided by ABA, among the most significant phytohormones, which regulates key transcriptional pathways. *AtARF2*, *AtARF3*, and miR390, *Arabidopsis* ARF transcription factors, contribute to diverse phytohormonal regulation through their downregulation [156].

## 5. Conclusions

miRNAs are a type of small non-coding RNA of 22 nucleotides in length and are considered critical gene expression regulators at the post-transcriptional stage. Various plant miRNAs are conserved across species. Such observations suggest that genetic changes based on miRNA in important crops can change environmental stress. The major function of miRNAs is the regulation of plant hormones. A complex network operates between protein-coding genes, phytohormones and miRNAs, regulating various plant development processes. They are significant key regulators for the proper development and growth of plants under both optimal and stress conditions, through phytohormone and miRNA-mediated gene expression regulation. In this review article, we compared the available miRNA literature with six phytohormone classes in crop development through genetic modulation of abiotic stress tolerance, including cytokinin (CK), gibberellic acid (GA), abscisic acid (ABA), ethylene (ET), auxin (AUX), and jasmonic acid (JA).

## Figures and Tables

**Table 1:** Phytohormones regulate the drought-responsive genes in various plant species.

Plant Hormone	Gene Regulation	Crop Name	Reference
IAA	Upregulation of drought-resistant TFs and genes	White clover	[33]
IAA, ABA, GA, BR	Upregulation of drought-responsive genes	Tea	[106]
CK	Overexpression of cytokinin oxidase genes	Tobacco	[31]
ET, ABA	Overexpression of ethylene-responsive factors (ERF9)	Tobacco	[107]
CK	Overexpression of cytokinin dehydrogenase genes	Barley	[108]
CK	Cytokinin biosynthesis gene *IPT*	Cotton	[63]
Aux, ABA, ET	Ectopic expression of *GhWOX4*	Cotton	[109]
ABA	Overexpression of *GhABF2*	Cotton	[110]
ABA	Overexpression of drought resistance genes *OsbZIP72*	Rice	[111]
ABA	Overexpression of drought resistance genes *OsbZIP46*	Rice	[40]
ABA, JA	Overexpression of *OsJAZ1*	Rice	[81]
ABA, JA	Overexpressing of *OsbZIP42*	Rice	[112]
ABA, GA, JA, IAA	Overexpressing of *OsSAP*	Rice	[113]
GA	Expression of GA2-specific mutants	Rice	[89]
ET	Expression of *OsERF109*	Rice	[114]
ABA	Ectopic expression of *OsSAPK2*	Rice	[115]
ET	Overexpression of ethylene response factors (ERFs)	Tomato	[116]
GA, ET	Downregulate the expression of *SlDREB*	Tomato	[117]
ABA	Upregulation of *SlGRAS4*	Tomato	[118]
BR	Upregulation of brassinosteroid biosynthetic gene *DWF4*	Brassica	[97]
ABA	Upregulation of *LOS5/ABA3*	Soybean	[119]
SA, ABA, GA	Overexpression of *GmTGA15* TFs	Soybean	[17]
ABA	Ectopic expression of *CaGol*	Chickpea	[120]
ABA, BR	Expression of *AtCAMTA1*	*Arabidopsis*	[121]

**Table 2:** miRNAs response to drought stress via phytohormonal signaling.

miRNA	Gene/Trait Effected	Hormone Involved	Plant Species	Reference
miR165	Drought tolerance	ABA	*Arabidopsis*	[160]
miR166	Drought stress tolerance	ABA	*Arabidopsis*	[160]
miR160	Leaf development	Aux	*Arabidopsis*	[160]
miR167	ARF6, AFR8	Aux	*Arabidopsis*	[77]
miR398	Upregulation	Aux	Tomato	[134]
miR952	Overexpression	Aux	Tomato	[134]
miR155	ROS homeostasis	ABA	Millet	[21]
miR156	Antioxidant enzymatic activities	ABA	Millet	[21]
miR399	9-cis-epoxycarotenoid dioxygenase1 proteins	ABA	Millet	[21]
miR164	9-cis-epoxycarotenoid dioxygenase1 proteins	ABA	Millet	[21]
miR444d	IF3 genes	ABA	Wheat	[161]
miR169d	ABA-responsive TFs	ABA	Wheat	[161]
miR156	Biosynthesis of anthocyanin genes	ABA		[162]
miR172	Upregulate AP2 TFs		Safflower	[19]
miR398	Detoxification of ROS		Safflower	[19]
miR164-MYB	Module drought stress	ABA	Maize	[163]
miR164-NAC	Module drought stress	ABA	Maize	[163]
miR159	Proline accumulation		Tomato	[164]
miR167	Downregulated	ABA	Rice	[142]
miR162	Slightly downregulated	ABA	Rice	[142]
miR413	Upregulated	ABA	Rice	[142]
miR166	Downregulated	GA	Rice	[142]
miR319	Downregulated	GA	Rice	[142]

## Data Availability

All the data related to this study are presented in the main text.
